# Fatigue, sleepiness and sleep quality are SARS-CoV-2 variant independent in patients with long COVID symptoms

**DOI:** 10.1007/s10787-023-01190-4

**Published:** 2023-04-05

**Authors:** Anna Reka Percze, Alexandra Nagy, Lorinc Polivka, Eniko Barczi, Ibolya Czaller, Zsuzsanna Kovats, Janos Tamas Varga, Judit H. Ballai, Veronika Muller, Gabor Horvath

**Affiliations:** Department of Pulmonology, Semmelweis University, Tomo u. 25-29., Budapest, 1083 Hungary

**Keywords:** Sleep quality, SARS-CoV-2, Variants of concern, Long COVID, Fatigue, Sleepiness

## Abstract

Acute infections with SARS-CoV-2 variants of concerns (VOCs) differ in clinical presentation. Discrepancies in their long-term sequelae, commonly referred to as long COVID, however, remain to be explored. We retrospectively analyzed data of 287 patients presented at the post-COVID care of the Pulmonology Department, Semmelweis University, Budapest, Hungary, and infected with SARS-CoV-2 during a period of 3 major epidemic waves in Hungary (February–July 2021, VOC: B.1.1.7, Alpha, N = 135; August–December 2021, VOC: B.1.617.2, Delta, N = 89; and January–June 2022, VOC: B.1.1.529, Omicron; N = 63), > 4 weeks after acute COVID-19. Overall, the ratio of long COVID symptomatic (LC) and asymptomatic (NS) patients was 2:1. Self-reported questionnaires on fatigue (Fatigue Severity Scale, FSS), sleepiness (Epworth Sleepiness Scale, ESS) and sleep quality (Pittsburgh Sleep Quality Index, PSQI) showed higher scores for LC (4.79 ± 0.12, 7.45 ± 0.33 and 7.46 ± 0.27, respectively) than NS patients (2.85 ± 0.16, 5.23 ± 0.32 and 4.26 ± 0.29, respectively; p < 0.05 for all vs. LC). By comparing data of the three waves, mean FSS and PSQI scores of LC patients, but not ESS scores, exceeded the normal range in all, with no significant inter-wave differences. Considering FSS ≥ 4 and PSQI > 5 cutoff values, LC patients commonly exhibited problematic fatigue (≥ 70%) and poor sleep quality (> 60%) in all three waves. Comparative analysis of PSQI component scores of LC patients identified no significant differences between the three waves. Our findings highlight the importance of concerted efforts to manage both fatigue and sleep disturbances in long COVID patient care. This multifaceted approach should be followed in all cases infected with either VOCs of SARS-CoV-2.

## Introduction

Coronavirus disease 2019 (COVID-19) caused by the severe acute respiratory syndrome coronavirus 2 (SARS-CoV-2) is still a major public health challenge all over the world. Its wide clinical spectrum ranges from asymptomatic infection to fatal disease. Nearly 3 years after the first reported case in Wuhan, China, SARS-CoV-2 continues to circulate globally, and leads to morbidity and mortality at an unprecedented scale (WHO 2022). Its emerging new variants of concern (VOC), such as the Alpha (B.1.1.7), Delta (B.1.617.2) and recently Omicron (B.1.1.529), impose further difficulties on effective diagnostic, treatment and prevention measures of SARS-CoV-2 infection (Sarkar and Madabhavi 2022).

Most people typically recover within a few weeks after the acute course of COVID-19, however, some may experience long-term post infection effects commonly referred to as “post-COVID-19 conditions” or “long COVID”. While a consensus upon the nomenclature and definition is still lacking, the National Institute for Health and Care Excellence (NICE) refers to long COVID as ongoing symptomatic COVID‐19 from 4 to 12 weeks, and to post‐COVID‐19 syndrome when signs and symptoms are persisting for more than 12 weeks after infection (NICE 2022). Long COVID is a heterogeneous condition occurring irrespectively of the initial severity of acute infection, and includes persistent or newly developed symptoms following acute COVID-19. Among others, these commonly include fatigue, cough, shortness of breath, sleep problems and cognitive dysfunction (Crook et al. 2021). Symptoms seem to be more prevalent in women and older patients. Although vaccinated people are less likely to develop the disease, the efficacy of vaccination remains to be elucidated. In addition to negative health, model calculations suggest a major role for long COVID related disability and organ damage in the burden of COVID-19 (Smith 2022).

The global prevalence of sleep disturbances during the COVID-19 pandemic is 30–40%, with the highest rates in patients with COVID-19 infection (52–75%) (Scarpelli et al. 2022). Sleep disturbances appears to be more prevalent amongst the more severe COVID-19 cases, and may persist for months following recovery from the acute disease (Davis et al. 2021). Despite the increasing body of literature, the role of sleep disturbances in long COVID is poorly understood. The recent International Covid Sleep Study-II (ICOSS-II) identified long-lasting sleep symptoms as core post-acute sequelae by showing high prevalence for fatigue (61.3%), insomnia symptoms (49.6%) and excessive daytime sleepiness (35.8%) in patients with long COVID symptoms (Merikanto et al. 2023). However, the importance of the type of viral variant (i.e. VOC) and its association with long COVID symptoms, such as fatigue and sleep problems, remains to be fully explored (Spinicci et al. 2022). Furthermore, the potential impact of the vaccination on the pattern of long COVID symptoms (“fenotype”) is not know.

In this study, we investigated fatigue, sleepiness and sleep quality in long COVID patients who were infected with SARS-CoV-2 during either the 3rd, 4th or 5th major epidemic waves of COVID-19 in Hungary. Since these waves are known to be dominated by Alpha, Delta and Omicron VOCs, respectively (Kiss et al. 2022; Voko et al. 2022), we aimed to understand the unique characteristics of these novel viral variants.

## Materials and methods

### Study population and procedures

**Fig. 1:**
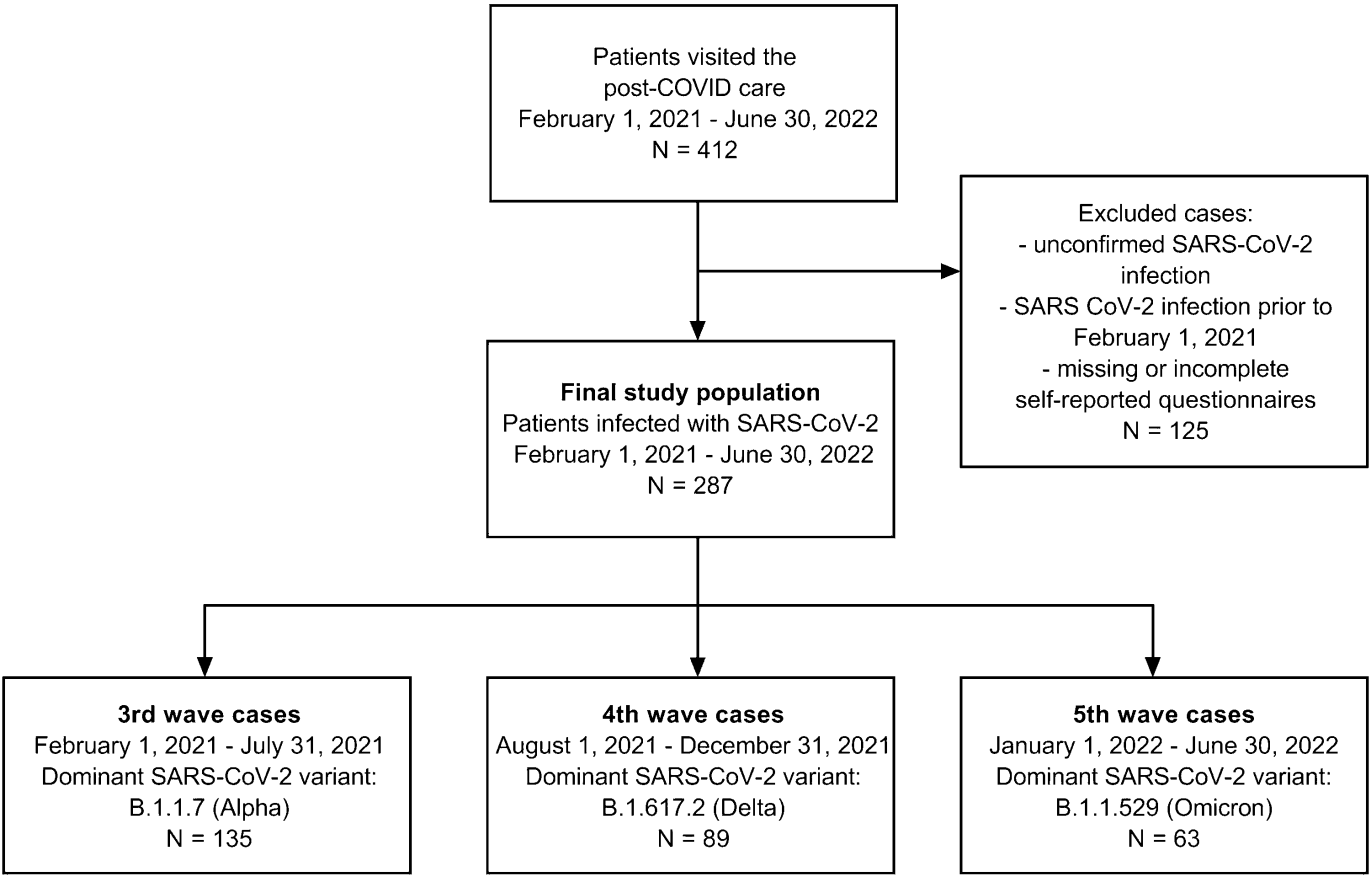
Study population

Our study is based on the retrospective review of 412 cases who were referred to the post-COVID care of the Semmelweis University, Department of Pulmonology between February 2021 and June 2022. A total of 287 cases met the following inclusion criteria: (1) acute COVID-19 during either the 3rd (between February 2021 and July 2021; VOC: B.1.1.7, Alpha; N = 135), 4th (between August 2021 and December 2021; VOC: B.1.617.2, Delta; N = 89) or 5th (between January 2022 and June 2022; VOC: B.1.1.529, Omicron; N = 63) major epidemic waves which affected Hungary (Voko et al. 2022; Kiss et al. 2022), (2) diagnosis confirmed by SARS-CoV-2 polymerase chain reaction (PCR) or a rapid immunoassay test, (3) presentation > 4 weeks after the acute course of COVID-19, and (4) complete medical records on anthropometric and self-reported questionnaire data (Fig. 1).

For statistical analysis, cases were categorized into two groups by expert respiratory physicians as cases with long COVID symptoms (LC group) and asymptomatic cases with clinically resolved COVID-19 (NS group). Categorization was based on the presence of one or more COVID-19 associated symptoms, such as cough, fatigue, dyspnoea, muscle pain, sleep problems, headache, palpitation, loss of taste or smell, sore throat, nasal congestion, nausea, fever and diarrhoea. Data analysis included records of self-reported questionnaires with special focus on fatigue, sleepiness and sleep quality.

The Fatigue Severity Scale (FSS), a 9-item self-report scale, was used to measure fatigue severity. Answers on how much fatigue affects the person’s activities and lifestyle are scored on a seven point scale where 1 = strongly disagree and 7 = strongly agree. FSS score is calculated as the mean of items’ scores, with higher scores indicating greater fatigue severity. The cutoff for problematic fatigue was set at an FSS score ≥ 4 as originally reported (Krupp et al. 1989).

The Epworth Sleepiness Scale (ESS) was used to assess daytime sleepiness. The questionnaire asks the subject to rate the probability of falling asleep in eight different situations on a scale of 0 to 3, and the item scores are summed to calculate ESS score. The cutoff score for increased sleepiness was set at an ESS score > 10 (Johns 1991).

The Pittsburgh Sleep Quality Index (PSQI), the most commonly used self-reported questionnaire, was applied to measure sleep quality (Buysse et al. 1989). PSQI assesses overall sleep quality during the previous month. The index comprises 19 items, and contains 7 subscales on subjective sleep quality, sleep onset latency, sleep duration, sleep efficiency, sleep disturbances, hypnotic medication use, and daytime dysfunction. Component scores, range from 0 to 3, are summed to calculate a global PSQI score. Higher scores indicate worse sleep quality. PSQI global score > 5 was considered as poor sleep quality (Takacs et al. 2016).

### Statistical analysis

Continuous variables are expressed as mean ± standard error of the mean (SEM). Normality of the data was determined using Kolmogorov–Smirnov test. Differences between groups for continuous data were evaluated in normally distributed data with Student’s t-test; otherwise, Mann–Whitney U-test was used. Chi-squared test was applied for comparing categorical variables. All percentage values are expressed for respective subgroups as indicated. For comparison between the waves of different VOCs, we used ANOVA (analysis of variance) with Tukey’s post hoc test. A p-value < 0.05 was defined as statistically significant. All analysis was performed using the GraphPad software (GraphPad Prism 5.0 Software, Inc., La Jolla, CA, United States) and SPSS v25 (IBM Corporation, Armonk, NY, United States).

## Results

The patient characteristics are summarized in Table 1. The study population included more males than females by about 35%. The mean age was nearly 54 years, whereas the mean body mass index (BMI) was in the overweight (25–29.9 kg/m^2^) range. Medical history analysis revealed hypertension as the most common (46.3%) comorbidity, followed by chronic respiratory diseases, other forms of cardiovascular diseases and diabetes. Evaluation of symptoms demonstrated that 189 patients still exhibited one or more long COVID symptoms (LC), whereas 98 patients were characterized as clinically resolved, asymptomatic COVID-19 cases (NS).

**Table 1:** Characteristics of patients with *(LC)* and without *(NS)* long COVID symptoms

	All patients N = 287	3rd wave N = 135		4th wave N = 89		5th wave N = 63	
		LC	NS	LC	NS	LC	NS
Age (years)	53.9 ± 0.9	54.2 ± 1.3	55.4 ± 2.1	50.1 ± 2.1	55.6 ± 3.3	55.7 ± 2.7	53.7 ± 3.3
Gender, *N* (%)							
Male	165 (57.5)	43 (48.9)	33 (70.2)	30 (50)	22 (75.9)	21 (51.2)	16 (72.7)
Female	122 (42.5)	45 (51.1)	14 (29.8)	30 (50)	7 (24.1)	20 (48.8)	6 (27.3)
BMI (kg/m^2^)	29.3 ± 0.4	29.9 ± 0.7	29.0 ± 0.7	30.1 ± 1.1	28.9 ± 1.0	27.6 ± 0.9	29.3 ± 1.3
Comorbidities, *N* (%)							
Hypertension	133 (46.3)	39 (44.3)	26 (55.3)	23 (38.3)	14 (48.3)	23 (56.1)	8 (36.4)
Chronic respiratory disease	59 (20.6)	20 (22.7)	2 (4.3)	13 (21.6)	5 (17.2)	12 (29.3)	7 (31.9)
Cardiovascular disease^a^	48 (16.7)	11 (12.5)	7 (14.8)	9 (15)	8 (27.6)	8 (19.5)	5 (22.7)
Diabetes	42 (14.6)	6 (6.8)	8 (17)	8 (13.3)	2 (6.9)	16 (39)	2 (9.1)
Thromboembolic disease	13 (4.5)	5 (5.7)	2 (4.3)	2 (3.3)	2 (6.9)	0 (0)	2 (9.1)

Data are presented as the mean ± SEM

*BMI* body mass index

^a^Cardiovascular disease other than hypertension

By analyzing data in each epidemic wave, mean age or BMI values of LC and NS groups showed no significant differences. The male:female ratio was 1:1 for LC groups, whereas close to 3:1 for NS groups in all waves (the reason for higher number of males in NS groups is not known). Comorbidity data are also shown for each wave in Table 1.

Concerning fatigue, sleepiness and sleep for the whole length of the 3 epidemic waves, mean scores of FSS, ESS and PSQI were 4.79 ± 0.12, 7.45 ± 0.33 and 7.46 ± 0.27, respectively for LC patients, significantly higher than for NS patients (2.85 ± 0.16, 5.23 ± 0.32 and 4.26 ± 0.29, respectively; p < 0.05 for all vs. LC). By considering the standard cutoff levels of FSS, ESS and PSQI, 71.3%, 24.5% and 62.8% of LC patients revealed problematic fatigue, increased sleepiness and poor sleep quality, respectively. For NS patients, these scores were 26.3%, 7.4% and 23.1%, respectively (p < 0.05 for all vs. LC).

**Fig. 2:**
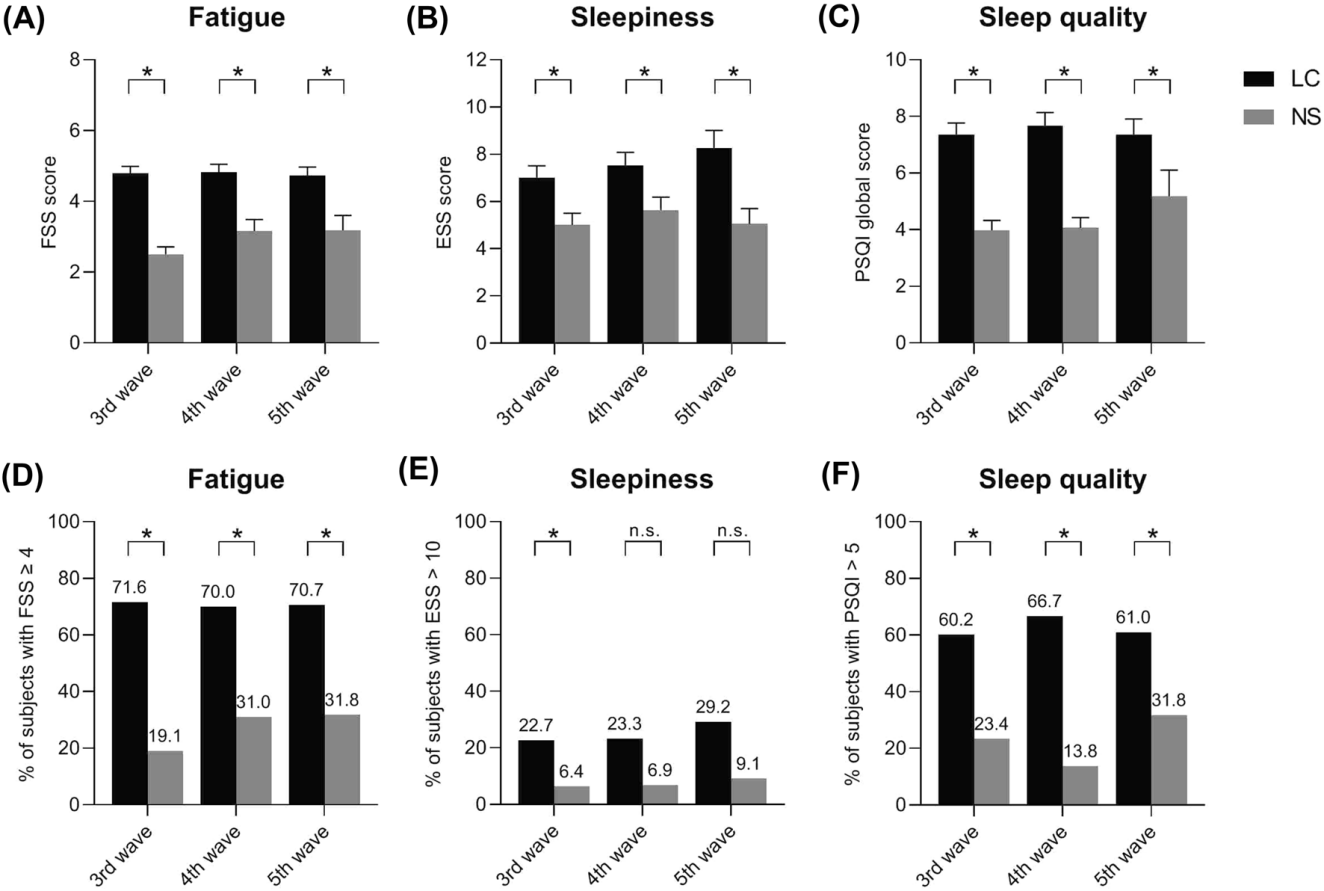
Fatigue (**A**, **D**), sleepiness (**B**, **E**) and sleep quality (**C**, **F**) in patients with (LC) and without (NS) long COVID symptoms. Fatigue Severity Scale (FSS), Epworth Sleepiness Scale (ESS) and Pittsburgh Sleep Quality Index (PSQI) data are presented as mean ± SEM on *Figure ***A**–**C**, whereas *Figure ***D**–**F** illustrate the percentage of subjects exceeding the standard cut-off values. See Table 1 for *n* values. n.s.: not significant, *p < 0.05

To assess unique characteristics of predominant VOCs in the 3 epidemic waves, the differences between fatigue, sleepiness and sleep quality data of 3 the waves were analyzed (Fig. 2). In all 3 waves, FSS mean scores exceeded the normal range for LC groups, but showed no significant inter-wave differences (Fig. 2A). In addition, FSS mean scores were significantly higher, and the prevalence of problematic fatigue was about three times higher for LC than NS groups in all three waves (Fig. 2A and D).Concerning sleepiness (Fig. 2B and E), ESS mean scores were in the normal range, although significantly higher numerically for LC than NS groups, in all three waves. In connection with sleep quality, PSQI mean scores exceeded the normal range for LC groups in all waves, which increase was also seen for NS group in the 5th wave (Fig. 2C). PSQI mean scores were significantly higher, and the prevalence of poor sleep quality was 2–4 times higher for LC than NS groups in all waves (Fig. 2C and F). However, PSQI scores showed no significant inter-wave differences.

**Fig. 3:**
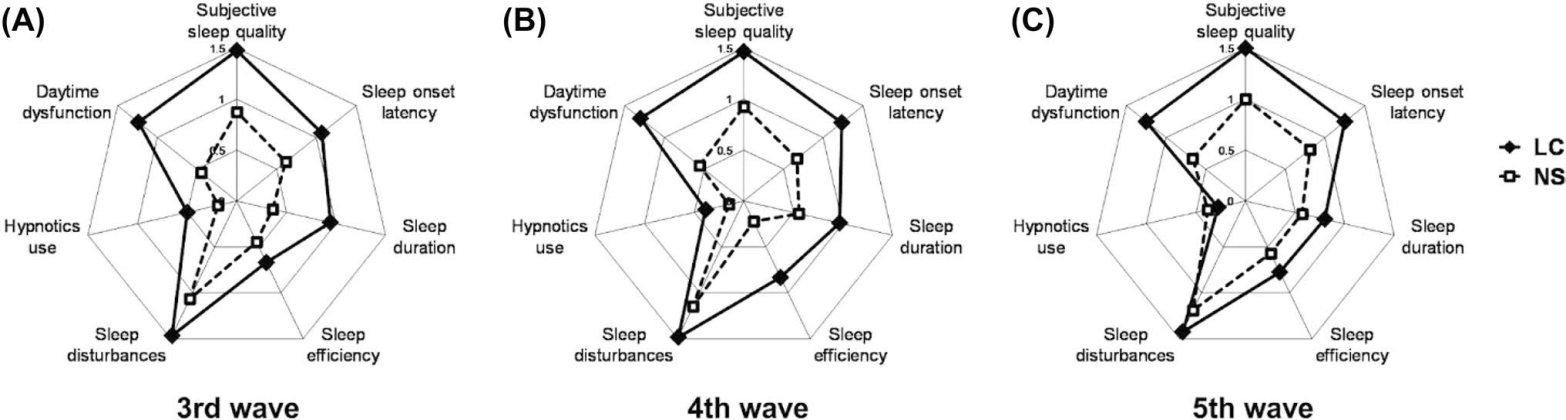
Radar plots showing sleep quality profiles of patients who were infected with SARS-CoV-2 during the 3rd (**A**), 4th (**B**) and 5th waves (**C**) of COVID-19. Markers show means of Pittsburgh Sleep Quality Index (PSQI) component scores in patients with (LC) and without (NS) long COVID symptoms. See Table 1 for *n* values

To further reveal possible unique characteristics of the three waves, the differences between PSQI component scores, each assessing a specific feature of sleep, were analyzed. Corresponding component scores for LC or NS groups were not significantly different in the three waves (Fig. 3). In the 3rd and 4th waves, PSQI component scores were significantly higher for LC than NS groups (Fig. 3A and B). However, as a minor difference in the 5th wave, component scores were significantly higher in three categories only (“Subjective sleep quality”, “Sleep onset latency” and “Daytime dysfunction”), but not in others (“Sleep duration”, Sleep efficiency”, “Sleep disturbances” and “Hypnotics use”) for LC than NS groups (Fig. 3C).

## Discussion

Recent evidence has shown that a number of complaints might persist long after the SARS-CoV-2 infection, but the virus variant-specific patterns of symptoms are not known. This retrospective study provides novel information on these “long COVID fenotypes” in connection with 3 VOCs, namely Alpha, Delta and Omicron. The main finding of the study is that, despite their differences between their clinical course of acute infection, these VOCs share important similarities in their long-term negative effects on fatigue and sleep quality.

Since the start of the COVID-19 pandemic, a number of VOCs has evolved resulting in multifarious clinical manifestations from asymptomatic disease to catastrophic somatic complications in the acute phase. With the introduction of vaccination, there is a major reduction in severe acute cases (WHO 2022; Voko et al. 2022; Kiss et al. 2022), while handling of long-term complications remains a challenging issue (Merikanto et al. 2023). Our study focused on VOCs of the last three consecutive epidemic waves of COVID-19. As far as our knowledge, these are the first Hungarian data on COVID-19 associated long-term complications, such as fatigue and sleep disturbances. Furthermore, this is the first study to assess the importance of 3 VOCs in association with long COVID symptoms.

Several studies have documented the fact that fatigue is a major manifestation of long COVID (Crook et al. 2021). Our result revealed high prevalence (~ 70%) of fatigue that was also confirmed by others recently (Baum et al. 2022). Whereas a slightly lower value (61.3%) was reported for severe cases in the recent ICOSS-II survey (Merikanto et al. 2023), the finding could be attributed to a distinct survey technique, and that only fatigue lasting at least 3 months was considered. It is also important to note the evolving vaccination status of patients that could influence individual long-term response to viral infections (Voko et al. 2022). The pathogenesis of fatigue can involve a variety of inflammatory, neurologic, metabolic, peripheral muscle, psychological and social factors in long COVID. In addition, certain other acute infections are also associated with post-acute infection syndromes, similar to the unexplained chronic disability in myalgic encephalomyelitis/chronic fatigue syndrome (Kedor et al. 2022), and may involve common underlying mechanisms.

This study was one of the first to comparatively measure fatigue and sleep quality in a prolonged 17-month period in a patient population affected by three different VOCs. Our study approach was supported by the evident discrepancies in clinical presentations of acute infections caused by different VOCs (Menni et al. 2022), and the suggested diversity in viral-host interactions. The hypothesis was evaluated in long COVID patients characterized by highly similar demographic and clinical features. We found striking similarities between the long-term effects of the 3 VOCs on fatigue and sleep quality. This notion was further reinforced by our comparative sleep quality analysis of PSQI component scores (i.e. each assess a specific feature of sleep) by revealing similar long-term responses to viral infection in all assessed aspects of sleep in the three waves. In addition to our observation on the 3 VOCs, since a uniform long-term effects were observed on both fatigue and sleep quality, our data could even support a common viral-host mechanistic pathway for chronic fatigue and sleep disturbances in long COVID.

Poor sleep quality was common (> 60%) among long COVID patients in our study, that finding is consistent with other recent studies (Kalamara et al. 2022; Merikanto et al. 2023). Multiple factors could have a role in pathogenesis of sleep disturbances in long COVID (Crook et al. 2021). SARS-CoV-2 can infect the central nervous system inducing neuroinflammation with damaging long-term effects. In addition to direct viral infection, suggested underlying pathological changes engendering sleep disturbances include cerebrovascular changes, infection induced harm caused by autoimmunity and inflammation-mediated damage to the central nervous system. The latter even being a self-influencing mechanism, which could lead to a vicious cycle, as sleep disorders can induce neuroinflammation, blood–brain barrier disruption, and promoting entry of antigens and inflammatory mediators into the brain (Semyachkina-Glushkovskaya et al. 2021). On the other hand, the pandemic itself can have long-lasting effects on mental health and sleep habits. Lockdown, quarantine, fear of the disease, isolation, social distancing, inability to work, financial concerns and reduced exposure to daylight could lead to depression, anxiety, poor sleep quality and sleeplessness. As sleep deprivation could have negative impact on immune response, sleep disturbances were also proposed to increase the susceptibility to the virus. Despite an increasing body of literature, however, further research is needed to fully explore the association of sleep disturbances and long COVID.

The main limitation of our study includes the retrospective single-centre design and the lack of healthy controls. The variables of SARS-CoV-2 vaccine types, dosing and schedule may also influence our results that, therefore, should be interpreted with caution. Additionally, infection severity and treatments were not analyzed.

## Conclusions

Our data reveal that, in addition to the fatigue, sleep problems can remain an important burden for long COVID patients. Since sleep quality and circadian rhythmicity have a profound role in physiological and mental wellbeing, these findings highlight the importance of sleep quality assessments and a multi-disciplinary approach in long COVID patient care. Furthermore, despite the reported differences in the course of acute disease caused by various VOCs, equally negative long-term effects should be expected for either Alpha, Delta or Omicron variants of SARS-CoV-2 on fatigue and sleep quality. Further research is needed to identify the impact of vaccination on fatigue and sleep disturbances in long COVID patients.

## Data Availability

The original contributions presented in the study are included in the article; further inquiries can be directed to the corresponding author.
